# Automatic Reconstruction of Multi-Level Indoor Spaces from Point Cloud and Trajectory

**DOI:** 10.3390/s21103493

**Published:** 2021-05-17

**Authors:** Gahyeon Lim, Nakju Doh

**Affiliations:** 1 School of Electrical Engineering, Korea University, Seoul 02841, Korea; gahyeonlim@gmail.com; 2 TeeLabs, Seoul 02857, Korea; 3 Institute of Convergence Science, Korea University, Seoul 02841, Korea

**Keywords:** automatic 3D modeling, structured 3D reconstruction, multi-level building reconstruction, point cloud processing

## Abstract

Remarkable progress in the development of modeling methods for indoor spaces has been made in recent years with a focus on the reconstruction of complex environments, such as multi-room and multi-level buildings. Existing methods represent indoor structure models as a combination of several sub-spaces, which are constructed by room segmentation or horizontal slicing approach that divide the multi-room or multi-level building environments into several segments. In this study, we propose an automatic reconstruction method of multi-level indoor spaces with unique models, including inter-room and inter-floor connections from point cloud and trajectory. We construct structural points from registered point cloud and extract piece-wise planar segments from the structural points. Then, a three-dimensional space decomposition is conducted and water-tight meshes are generated with energy minimization using graph cut algorithm. The data term of the energy function is expressed as a difference in visibility between each decomposed space and trajectory. The proposed method allows modeling of indoor spaces in complex environments, such as multi-room, room-less, and multi-level buildings. The performance of the proposed approach is evaluated for seven indoor space datasets.

## 1. Introduction

Three-dimensional (3D) models of indoor spaces are widely used in the field of virtual reality (VR), augmented reality (AR), indoor localization, and indoor navigation. Modeling methods of the indoor structure are important topics of study in various fields, such as computer vision, computer graphics, civil engineering, and robotics. Nevertheless, the reconstruction of indoor structures that include inter-room connections and inter-floor connections is a challenge. A recent survey and tutorial indicated that modeling with inter-room and inter-floor connections approach is reserved for future work. Pintore et al. [1] presented the modeling of an entire building; however, the connections between rooms and levels is still a complex problem. Hence, a global solution is required to reconstruct complex environments [2].

Previous works address this problem based on divide-and-conquer techniques. Floor segmentation of buildings and room segmentation are traditional approaches that convert the difficult problem into simpler individual sub-problems for modeling of indoor spaces. The floor segmentation approach divides multi-level buildings into several single-level spaces, whereas the room segmentation approach divides single-level multi-room spaces into several rooms. Models of single-level indoor spaces are represented by a combination of models in individual room and that of multi-level buildings are represented by a combination of such single-level models.

**Figure 1:**
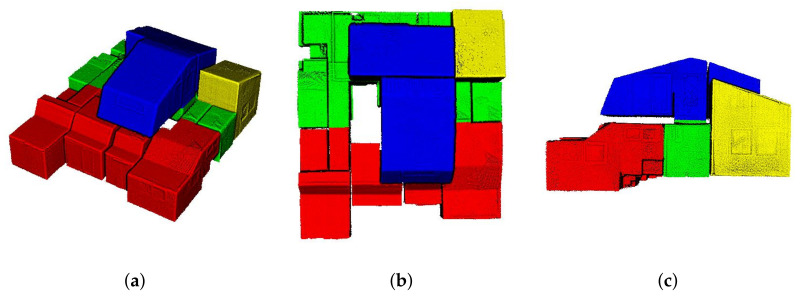
Registered point cloud of an indoor space with complex multiple floors environment (C2 in our dataset). (**a**) The bird’s-eye view, (**b**) top view, and (**c**) side view of the indoor space. The combination of first and 0.5 floors is denoted by red, first floor is denoted by green, second floor is denoted by blue, and a combination of first and second floors is denoted by yellow. The conventional horizontal slicing approach is not suitable for complex multi-level building environments.

Oesau et al. [3] introduced a reconstruction method for separating multi-level buildings into individual single-level spaces using horizontal slicing approach. The horizontal slicing algorithm was used to cut the target space using peaks in vertical histogram of point cloud distributions. However, this approach cannot be used to separate each level of complex multi-level buildings depicted in Figure 1, because the indoor space, which is C2 in our datasets, comprises multiple levels of varying height.

Some studies conducted room segmentation using Markov clustering [4,5,6] or space over-segmentation and merging [7,8,9]. These approaches may be suitable for modeling of multi-room environments but not for room-less environments.

**Figure 2:**
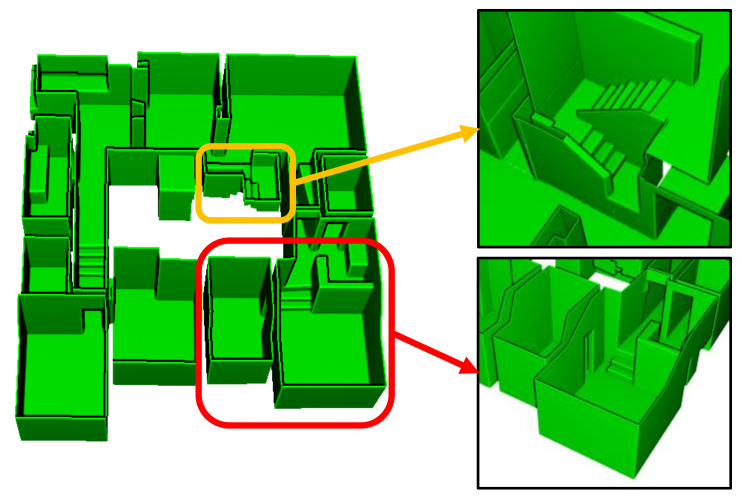
Mesh model built using our proposed method of indoor structure modeling includes inter-room (doors or openings) and inter-floor connections (stairs), denoted by the red and yellow boxes, respectively.

Furthermore, certain existing approaches still use the room segmentation for modeling multi-room environments, modelling methods for inter-room connections have not been addressed [10,11]. The inter-room and inter-floor connections can be modeled as doors (or openings) and stairs, respectively, as depicted in Figure 2. The figure illustrates the reconstructed model of the dataset C2 using the proposed method.

Previtali et al. [12] detected inter-room connections using lay-tracing algorithm, whereas Wang et al. [13] and Yang et al. [14] used specific parameters, such as upper and lower bound of doors or width of doors. Oesau et al. [3] dealt with the modeling of multi-level buildings but did not model inter-floor connections. This approach generated multi-level building models by stacking multiple single-level spaces. Nikoohemat et al. [15] divided the target multi-level building into floor and stair parts and constructed the multi-level building model as a combination of multiple single-level spaces and stair models.

In this paper, we propose an automatic reconstruction method for multi-level indoor spaces with complex environments. The proposed method can generate indoor space models that include inter-room and inter-floor connections from point cloud and trajectory. The continuous trajectory through multiple rooms and multiple floors within a building is used to reconstruct an indoor space as a unique model. Furthermore, the proposed method does not segment structural points into specific components such as walls, floors, ceilings, doors, or stairs, and does not segment indoor spaces into sub-spaces such as individual rooms or single-level floors. The proposed method provides a general approach to indoor space modeling that reconstructs indoor structures into piece-wise planar segments and builds the target indoor space to a unique model, even if the target indoor space consists of multiple rooms or multiple floors. In addition, the proposed method conducts energy minimization using graph cut, which enables automatic reconstruction of indoor space models. We validated the performance of the proposed approach by evaluating the error in the distance between point cloud and generated mesh for seven datasets. Furthermore, by considering datasets from various environments, this approach validates the wide applicability of the proposed method in multi-room, room-less, and multi-level building environments.

The proposed method is improved over our previous work [16]. Our previous work used random sample consensus (RANSAC) based plane extraction and constructed an adjacency graph to reconstruct indoor spaces, but this approach requires manual work when generating the adjacency graph. However, in this paper, the proposed method uses region-growing based plane extraction to detect small plane patches such as stairs, doors, or openings and automatically reconstructs indoor space models with 3D space decomposition and energy minimization using graph cut. Furthermore, the proposed method uses both point cloud and trajectory to build the indoor space of multi-level building as a unique model that includes inter-room and inter-floor connections. In particular, the data term of the energy function is expressed as a difference in visibility between each decomposed space and trajectory.

The remainder of this paper is organized as follows. Section 2 presents an overview of the existing literature. Section 3 describes the proposed method and discusses the construction of indoor space models, including inter-room and inter-floor connections from point cloud and trajectory. Section 4 presents the experimental results and performance evaluation of the proposed method for seven datasets. Section 5 presents the conclusion.

## 2. Related Work

Methods for modeling of indoor spaces are still actively studied in various fields, such as computer vision, computer graphics, civil engineering, and robotics. Existing methods build indoor space models using geometric information, images, or a combination of both. The modeling of indoor spaces from point cloud is the most traditional approach. However, it is optional to use a trajectory, the location of LiDAR scan raw data, in existing methods that use point cloud. Moreover, some studies use only point cloud [5,6], whereas some use both point cloud and information of scan position [4,17].

Many existing approaches use geometric assumptions to model 3D indoor spaces, such as the Manhattan world assumption or two-and-a-half-dimensional (2.5D) approaches. Ikehata et al. [18], Murali et al. [19], and Xie et al. [20] built models under the Manhattan world assumptions. Mura et al. [21], Ochmann et al. [17], and Wang et al. [13] introduced methods using 2.5D approaches that construct the models by vertical extension of two-dimensional (2D) floor plans. In the 2.5D environments, the walls are orthogonal to a single floor and ceiling. These previous works with 2.5D approaches first detected a floor and ceiling, then projected wall points onto the floor to generate floor plans in 2D.

Recently, 3D approaches have been introduced because the methods using geometric assumptions (i.e., the Manhattan world assumptions and 2.5D approaches) cannot reconstruct indoor spaces with complex environments. Some of the studies address fully 3D modeling approaches that can model more complex environments [4,6,10]. These approaches conduct 3D space decomposition using 3D planar segments extracted from the point cloud. Mura et al. [4] used binary space partitioning (BSP) to decompose 3D spaces. Ochmann et al. [6] and Nikoohemat et al. [10] reconstructed 3D models with volumetric walls.

An extraction of structural components is conducted to model real-world indoor spaces, which are cluttered environments. The geometric information (i.e., point cloud) of the target indoor space can be segmented into structural and object (non-structural) parts. The structural parts are architectural components of the indoor spaces, such as floors, ceilings, walls, and stairs. However, previous methods extracted structural parts without segmenting into structural and non-structural parts or used assumptions to extract structural parts. Previtali et al. [12] detected wall components under the Manhattan world assumption. Macher et al. [22] assumed that wall points are located in the boundary of the rooms to detect walls. Mura et al. [4] detected the permanent components using structural patterns. They assumed that the permanent components are rectangles so that the holes caused due to occlusion by objects can be neglected. Nikoohemat et al. [10] presented an adjacency graph based permanent structures detection method. Lim et al. [16] not only segmented structural and object points but also filled in the holes that are used to construct architectural points by projecting object points onto the structural surfaces. Coudron et al. [23] used deep learning to extract permanent structures.

Structural primitives (e.g., 2D lines or 3D piece-wise planar segments) can be extracted from the structural components using various plane detection algorithms. Xiong et al. [24] detected structural patches by region-growing algorithm based on plane detection using total least squares. Xiao et al. [25] used Hough transform to detect wall components in 2D. Turner et al. [26] segmented planar segments using principal component analysis. Tran et al. [11] **and Ochmann et al**. [6] used the RANSAC based plane-fitting algorithm [27] for extracting structural surfaces.

In indoor spaces with multi-room environments, the room segmentation approaches divide the modeling problems to several simpler sub-problems and are conducted using the Markov clustering algorithm [4,5,6] or space over-segmentation and merging approach [7,8,9]. The models of indoor spaces represent a combination of individual rooms divided by room segmentation. Yang et al. [14] and Mura et al. [4] conducted room segmentation in 2D and 3D models, respectively. Mura et al. [4] conducted the procedure by allocating separate rooms using the Markov clustering algorithm and built models through a multi-label energy minimization approach [28], but the modeling method of inter-room connections was not described. Yang et al. [14] detected inter-room connections using specific parameters, such as upper and lower bound of doors. Previtali et al. [12] and Wang et al. [13] introduced a modeling method through a two-label (*interior* and *exterior*) energy minimization approach under the Manhattan world assumption and 2.5D approaches, respectively. These approaches detected the inter-room connections using lay-tracing or reasonable parameters (i.e., width of doors).

Oesau et al. [3] introduced the horizontal slicing approach that separated multi-level buildings through the peak of horizontal (z-axis) histogram of the distribution of point cloud. Then, the models of multi-level buildings were represented as a combination of models for a single-level space. Additionally, this approach can reconstruct the room-less environments by implementing the 2.5D approaches. However, it cannot model the complex multi-level environments as depicted in Figure 1. Nikoohemat et al. [15] used a trajectory to segment the point cloud of a multi-level building. The segments of horizontal trajectory and sloped trajectory divided the indoor space into floor and stair parts. Then, the generated models were represented as a combination of floor and stair models.

In this paper, we propose the modeling method that does not use geometric assumptions (i.e., the Manhattan world assumption and 2.5D approach) and convert difficult modeling problems into several simpler sub-problems (i.e., horizontal slicing and room segmentation). Furthermore, the proposed method uses a trajectory through multiple rooms and multiple floors in a building to reconstruct indoor space model that include inter-room and inter-floor connections.

## 3. Methods

**Figure 3:**
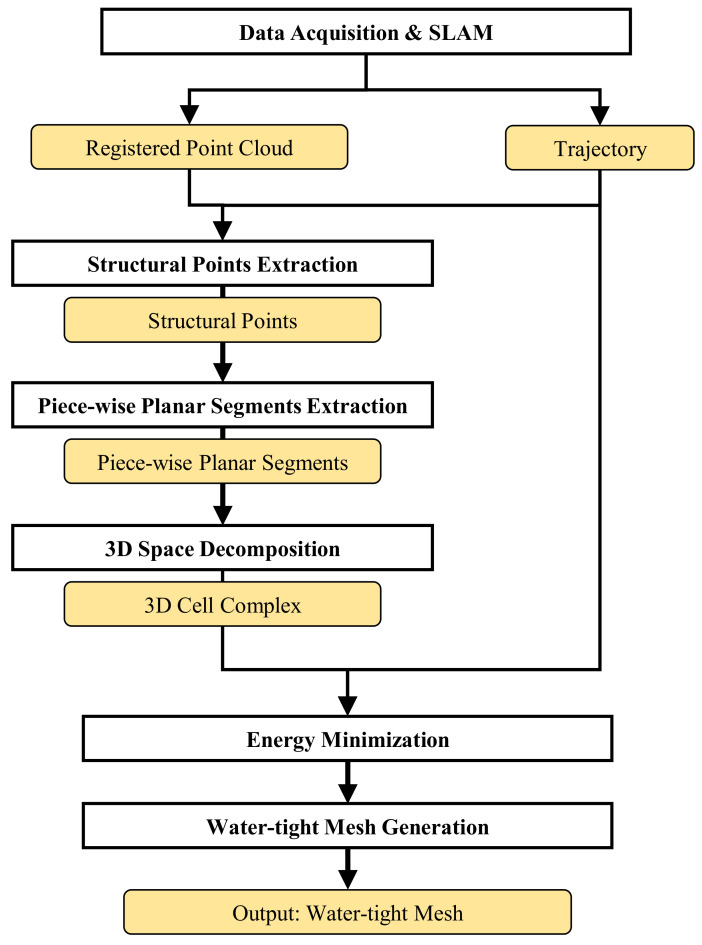
Flowchart of the proposed method.

This section describes the method to generate indoor structure mesh models that include inter-room and inter-floor connections from the point cloud and trajectory as depicted in Figure 3. First, we construct the structural points, which represents the architectural components of indoor spaces, from the registered point cloud and trajectory in the pre-processing step. Next, the proposed method extracts piece-wise planar segments to decomposes the indoor space into the 3D cell complex. Finally, water-tight meshes are generated through energy minimization using the graph cut algorithm. We use the trajectory to compute the difference in visibility in the energy function minimization.

### 3.1. Pre-Processing

**Figure 4:**
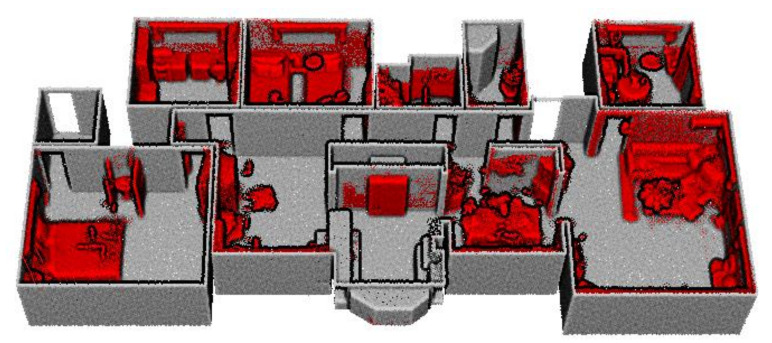
Structural points (grey) and the object points (red) that are segmented from the registered point cloud of our dataset A1.

We acquire the registered point cloud and trajectory, which are the optimized poses of raw LiDAR measurements, using the LiDAR-IMU based simultaneous localization and mapping (SLAM) [29,30]. The structural points are constructed using the architectural point cloud construction described in our previous work [16]. First, the registered point cloud is segmented into structural and non-structural components. Then, the structural points are constructed by projecting non-structural points onto adjacent piece-wise planar structural segments extracted from the structural components. Figure 4 demonstrates the constructed structural points (grey) and segmented object points (red) of our dataset A1.

### 3.2. Piece-Wise Planar Segments Extraction

This study is aimed at reconstructing indoor spaces including inter-room and inter-floor connections; hence, detailed planar segments are extracted using plane extraction algorithms. We conduct the region-growing based plane detection algorithm [31] in the computational geometry algorithms library (CGAL) [32]. This approach is suitable for extracting piece-wise planar segments in complex environments.

The piece-wise planar segments *P* are extracted in two steps. Large piece-wise planar segments are first extracted and small surfaces are detected from the remaining points not included in the pre-extracted piece-wise planar segments. The parameters for piece-wise planar segments extraction can be modified by varying noise or density of the structural points. Table 1 records the parameters usually used in our tests for piece-wise planar segments extraction. *k* denotes the number of nearest neighbors used to calculate the normal vector of the target points. $dist_{max}$ denotes the maximum distance from the plane to the points that consist the plane. $angle_{max}$ denotes the maximum angle between the two regions for merging. $N_{min}$ denotes the minimum number of points that consist piece-wise planar segments.

### 3.3. 3D Space Decomposition

**Figure 5:**
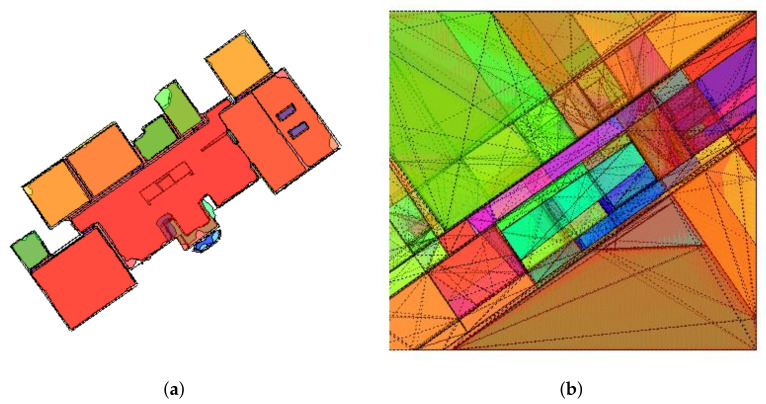
Top view of (**a**) piece-wise planar segments and (**b**) 3D cell complex of our dataset A1.

The 3D cell complex *C* (Algorithm 1.) is constructed by decomposing the indoor space into polyhedral cells using piece-wise planar segments. For efficient space decomposition, the cell *c* (c∈C) is divided into two cells by piece-wise plane segment *p* (p∈P) when the points of the piece-wise planar surface $X_{p}$ are inside the cell *c*. Figure 5 illustrates extracted piece-wise planar segments and 3D cell complex of our dataset A1.
**Algorithm 1** 3D cell complex construction1:C←Initialization(P)2:**for**i=1:n**do**3: **for** j=1:m **do**4:  **if** $X_{p_{i}}$ is inside $c_{j}$ **then**5:   $C\leftarrow Decomposition(C,c_{j},p_{i})$6:  **end if**7: **end for**8:**end for**

A visibility $Vis_{c}$ denotes the score that represents the visibility of piece-wise planar segments in the cell *c*, and adjacency Adj is the set of pairs of adjacent cells.

(1) $$Adj=\{\left(c,c^{\prime }\right)|c,c^{\prime }\in C\cap c,c^{\prime }\mathrm{are adjacent}.\}$$

$Vis_{c}$ is represented by the distribution of visible region of each piece-wise planar segments. Specifically, $visArea_{c}^{p}$ denotes the visible area of the piece-wise planar segment *p* in the cell *c*. As the structural points are sparse, simple ray-tracing based visible points detection algorithms can detect false points. Therefore, we propose visible points detection algorithm that is robust for a varied density of points, as shown in Algorithm 2. First, the structural points $X_{str}$ are transformed into spherical coordinate with the center of the cell as the origin. Next, the shortest distance from the origin to the transformed structural point is used to calculate the angle deviation (δψ) of the latitude and longitude to divide the space. Finally, the point closest to the origin is selected as a visible point in each region divided by the angle deviation. Because we conduct a 1 cm grid sampling on the point cloud of the experiments, we set the reference angle deviation to tan ${}^{-1}$(0.01) to detect the points sampled at 1 cm grid. The parameter *k* is set to 2 in our tests.
**Algorithm 2** Visible points detection in the cell1:$map\left(r,\theta ,\phi \right)\leftarrow ConvertCoordinate\left(X_{str},center\right)$2:$\delta \psi =k\;tan^{-1}\left(0.01/min\left(r\right)\right)$3:**for**i=1:δψ:2π**do**4: **for** j=1:δψ:π **do**5:  $X_{vis}\left(i,j\right)\leftarrow f\left(map,center,i,j,\delta \psi \right)$6: **end for**7:**end for**8:$X_{vis}=⋃X_{vis}\left(i,j\right)$

The visible points are segmented according to piece-wise planar segments, and the visible area of each piece-wise planar segments is calculated from the segmented visible points.

### 3.4. Water-Tight Model Reconstruction

We implement energy minimization using graph cut algorithm [28] with two labels, namely *interior* $L_{int}$ and *exterior*$L_{ext}$, to reconstruct the indoor spaces. The energy function to be minimized consisted of the data and smoothness terms expressed as:

(2) $$E\left(l\right)=\sum_{c\in C}D_{c}\left(l_{c}\right)+\lambda \sum_{\{c,c^{\prime }\}\in Adj}V_{c,c^{\prime }}\left(l_{c},l_{c^{\prime }}\right)$$

where $\sum_{c\in C}D_{c}\left(l_{c}\right)$ and $\sum_{\{c,c^{\prime }\}\in Adj}V_{c,c^{\prime }}\left(l_{c},l_{c^{\prime }}\right)$ represent the data and smoothness terms, respectively. $l_{c}$ is a label of the cell c∈C, and parameter λ is the ratio of weight of the data and smoothness terms. We used λ as a value of 0.1∼0.2 in our experiments.

#### 3.4.1. Data Term

The data term is constrained using difference in visibility between the 3D cell complex *C* and trajectory *T*:

(3) $$D_{c}\left(l_{c}\right)=\left\{\begin{array}{cc} \min_{t\in T}visDiff\left(c,t\right), & \mathrm{if}l_{c}\in L_{int}\\ 1-\min_{t\in T}visDiff\left(c,t\right), & \mathrm{if}l_{c}\in L_{ext} \end{array}\right.$$

(4) $$visDiff\left(c,t\right)=\frac{{\left(\frac{1}{n}\sum_{p=1}^{n}{\left(visArea_{c}^{p}-visArea_{t}^{p}\right)}^{2}\right)}^{\frac{1}{2}}}{{\left(\frac{1}{n}\sum_{p=1}^{n}\left(\max{\left(visArea_{c}^{p},visArea_{t}^{p}\right)}^{2}\right)\right)}^{\frac{1}{2}}}$$

**Figure 6:**
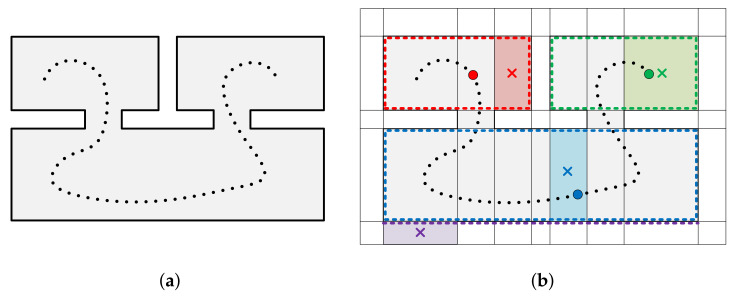
Illustration of an indoor space consisting of three rooms and two inter-room connections expressed in 2D for visualization. The (**a**) structural points (black lines) and trajectory points (black dots) and (**b**) decomposed 3D spaces. (**b**) illustrates centers of the cells (colored crosses), visible points in the cells (colored dots), and trajectory points with the smallest difference in visibility (colored circles) for each cell (colored boxes). The cells marked in red, green, and blue select the trajectory point with the smallest difference in visibility but a cell marked in purple, which is outside the indoor space, has large differences in visibility for all trajectory points.

Specifically, visDiff(c,t) is the difference in visibility between a cell *c* (c∈C) and a trajectory point *t* (t∈T). The $visArea_{c}^{p}$ and the $visArea_{t}^{p}$ are visible areas of a piece-wise planar segment p∈P from a cell c∈C and a trajectory point t∈T, respectively, using Algorithm 2 and 3. We calculate the difference in visibility for all trajectory points in each cell and select the trajectory point with the smallest value of visDiff(c,t). Figure 6 illustrates selected trajectory points in each cells. Cells inside the indoor spaces can select the trajectory points with smallest difference in visibility, whereas cells outside the indoor spaces have large differences in visibility for all trajectory points. The selected trajectory points are not the closest points from the corresponding center of cells because they are determined by the difference in visibility. In addition, cells located in regions of inter-room and inter-floor connections can be labeled *interior* because the trajectory is continuous through multiple rooms or multiple levels in buildings.
**Algorithm 3** Visible area detection in the cell1:**for**i=1:n**do**2: $X_{vis,c_{i}}\leftarrow$ VisiblePointsDetection $\left(X_{str},center_{c_{i}}\right)$3: **for** j=1:m **do**4:  $X_{vis,c_{i}}^{p_{j}}\leftarrow$ Segmentation($X_{vis,c_{i}}$,$p_{j}$)5:  $visArea_{c_{i}}^{p_{j}}\leftarrow$ VisibleAreaDetection($X_{vis,c_{i}}^{p_{j}}$)6: **end for**7:**end for**

#### 3.4.2. Smoothness Term

The smoothness term is defined as the ratio of the area occupied by the structural points located on the adjacent face between two adjacent cells:

(5) $$V_{c,c^{\prime }}\left(l_{c},l_{c^{\prime }}\right)=\left[1-w_{c,c^{\prime }}\right]\cdot g\left(l_{c},l_{c^{\prime }}\right)$$

(6) $$w_{c,c^{\prime }}=\frac{occupiedArea_{c,c^{\prime }}}{faceArea_{c,c^{\prime }}}$$

(7) $$g\left(l_{c},l_{c^{\prime }}\right)=\left\{\begin{array}{cc} 1, & \mathrm{if}l_{c}\neq l_{c^{\prime }}\\ 0, & \mathrm{if}l_{c}=l_{c^{\prime }} \end{array}\right.$$

**Figure 7:**
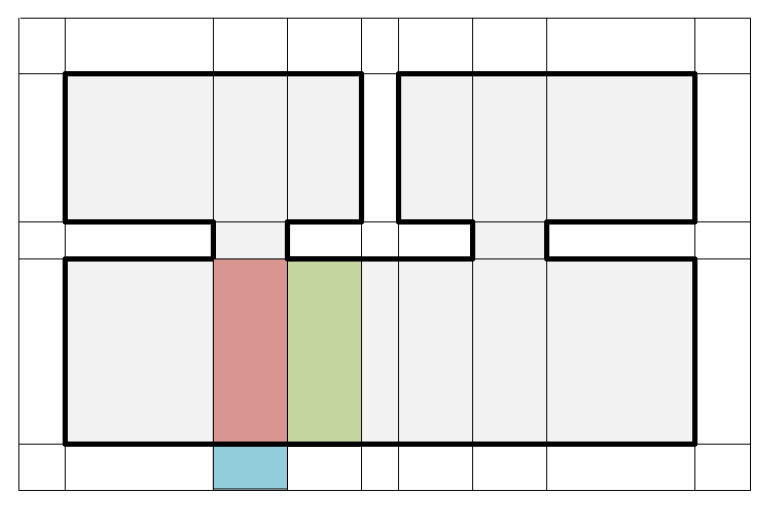
Illustration of two pairs of adjacent cells of an indoor space depicted in Figure 6a expressed in 2D for visualization. The area occupied by structural points located on the adjacent face of red and green cells is close to zero, while that of red and blue cells is close to the area of face shared by the two cells.

The $occupiedArea_{c,c^{\prime }}$ and $faceArea_{c,c^{\prime }}$ represent the area occupied by structural points located on the adjacent face of the two cells ($c,c^{\prime }$) and area of the face shared by the two adjacent cells, respectively ($occupiedArea_{c,c^{\prime }}\leq faceArea_{c,c^{\prime }}$). The $occupiedArea_{c,c^{\prime }}$ is close to zero when the cells (c, c’) are located both inside or outside of indoor spaces, whereas that is close to the $faceArea_{c,c^{\prime }}$ when the cells are located different region (inside or outside) of indoor spaces. Therefore, the cells with low $w_{c,c^{\prime }}$ are connected to large weights on a graph, while the cells with high $w_{c,c^{\prime }}$ are connected to low weights on the graph. Figure 7 illustrates two pairs of adjacent cells depicted red and green cells and red and blue cells.

#### 3.4.3. Water-Tight Mesh Generation

The indoor space models are generated as boundary polyhedral of the set of interior cells labeled $L_{int}$. The models are represented by triangular meshes via 2D constrained Delaunay triangulation algorithm [33] for each target face of cells.

## 4. Experimental Results

In this section, we validated the proposed method by evaluating its performance for seven datasets, with five real-world indoor spaces and two public datasets.

### 4.1. Dataset

**Figure 8:**
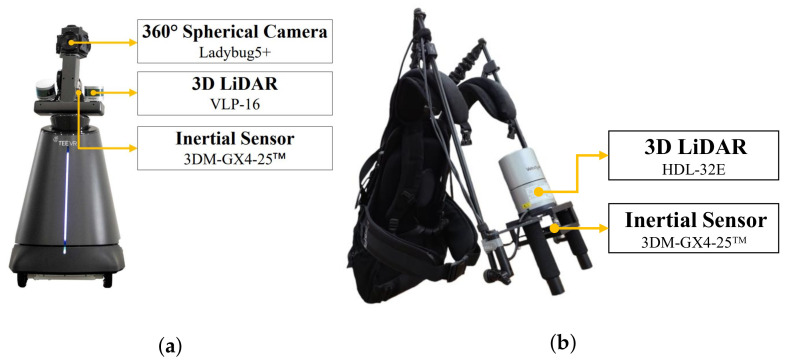
(**a**) Robot system and (**b**) backpack system employed for data acquisition.

We collected the data on real-world indoor spaces using a robot system and a backpack system as illustrated in Figure 8. The robot system is a combination of a spherical camera, two 3D LiDARs, and an inertial sensor, while the backpack system consists of a 3D LiDAR and an inertial sensor. The registered point cloud and trajectory of the real-world indoor spaces were generated by LiDAR-IMU based SLAM [29,30].

For efficient contextualization, datasets are classified according to their environmental characteristics:

- Group A: Environments with multiple rooms and a single level.
- Group B: Environments with no room and a single level.
- Group C: Environments with multiple rooms and multiple levels.

Table 2 records the environmental information of the indoor spaces used in the experiment. A1, B1, B2, C2, and C3 are the real-world indoor space datasets acquired by the robot or backpack systems. A2 is the “TUB1” on the ISPRS benchmark datasets [34] and C1 is the “house” on the UZH rooms detection datasets [35]. In Table 2, the “#Registered points” and “#Trajectory points” indicate the number of registered point cloud and optimized pose of raw LiDAR scan locations generated by SLAM, respectively. The “#Rooms”, “#Floors”, and “#Stairs” indicate the number of rooms, floors, and stairs in the target spaces, respectively.

**Figure 9:**
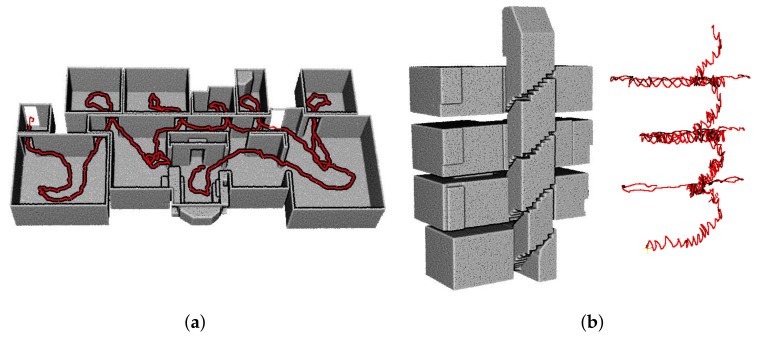
Structural points (grey) and the trajectory points (red) of (**a**) dataset A1 and (**b**) dataset C3. The trajectory is continuous through multiple rooms or multiple levels.

Figure 9 demonstrates the structural points and trajectory points of the datasets A1 and C3. The trajectory of the dataset A1 shown in Figure 9a is continuous through multiple rooms, and the trajectory of the dataset C3 shown in Figure 9b is continuous through multiple levels in the building.

### 4.2. Results of Structural Points Extraction

The registered points can be segmented into structural points, non-structural (object) points, and noise points. Structural points are extracted using the architectural point cloud construction approach in [16]. In Table 3, the “#Registered points” and “#Structural points” indicate the number of registered point cloud and structural points, respectively.

### 4.3. Results of Model Generation

**Figure 10:**
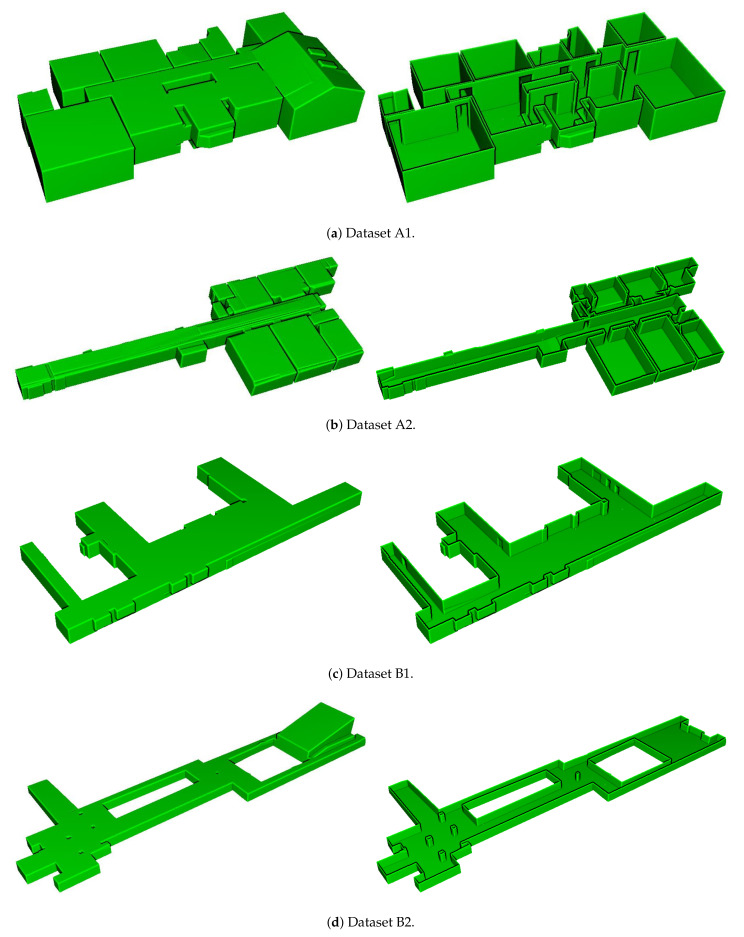

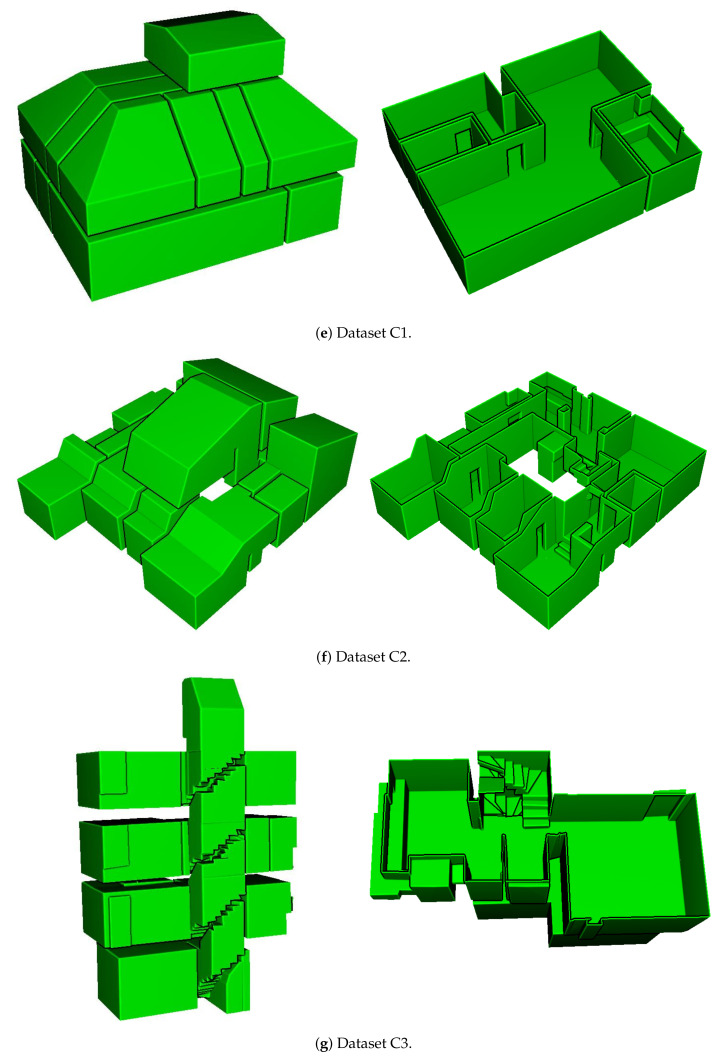
Results of the generated water-tight meshes. The bird’s-eye view of the entire spaces (**left**) and the fist floor without ceilings (**right**) are displayed.

The generated indoor structure mesh models are illustrated in Figure 10, which demonstrates that the proposed method successfully modeled the entire datasets, including environments with multi-room (A1, A2), room-less (B1, B2), and multi-level buildings (C1, C2, C3). In particular, the environments with slanted structures (A1, B2, C1, C2, C3) were modeled. In addition, the inter-room and inter-floor connections were reconstructed as shown in (A1, A2, C1, C2, C3) and (C2, C3), respectively. Note that because the dataset C1 did not provide sufficient point cloud and trajectory for stairs, we did not build models of stairs.

**Figure 11:**
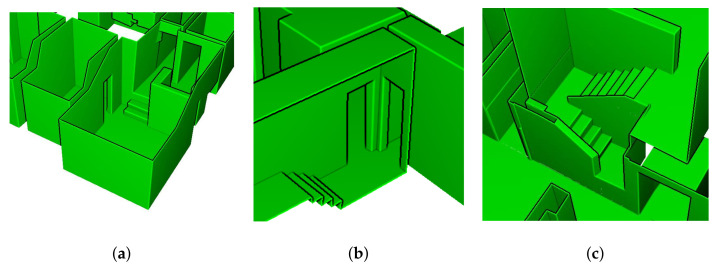
Detailed images of dataset C2. (**a**) inter-floor and three inter-room connections, (**b**) inter-floor and two inter-room connections, and (**c**) inter-floor connection.

**Figure 12:**
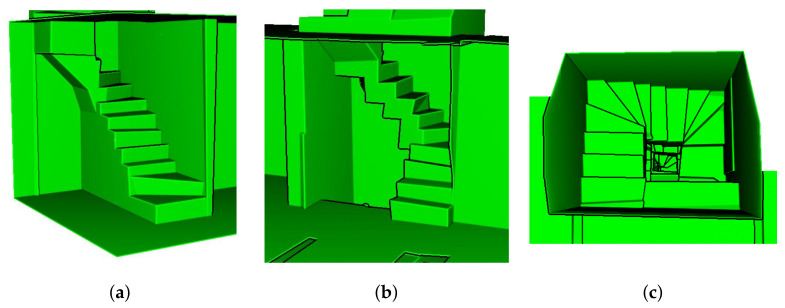
Detailed images of dataset C3. (**a**) inter-floor connection between underground and first floor, (**b**) inter-floor connection between first and second floor, and (**c**) top view of inter-floor connection between third and fourth floor.

The detailed illustrations of the modeling results of inter-floor and inter-room connections for dataset C2 and C3 are presented in Figure 11 and Figure 12, respectively, which convey that the inter-floor (stairs) and inter-room connections (doors or openings) were successfully modeled using the proposed approach.

The accuracy of the reconstructed indoor structure mesh models were evaluated by the error in distance between the structural points and corresponding meshes. Table 4 lists the median distance error and median absolute deviation (MAD) [36] of the datasets.

## 5. Conclusions and Future Work

**We proposed a method for automatic reconstruction of indoor structure comprising** inter-room and inter-floor connections in multi-level buildings from point cloud and trajectory. The proposed method allows the modeling of multi-room environments with inter-room connections, room-less environments, and multi-level buildings with inter-floor connections. We constructed a structural points from the registered point cloud. Then, piece-wise planar segments were extracted to decompose the indoor spaces. Finally, water-tight meshes of indoor spaces were generated through energy minimization using graph cut algorithm. The trajectory through multiple rooms and multiple floors within the buildings was used to determine the difference in visibility in the data term of an energy function. Experimental results for seven datasets were recorded demonstrating that the proposed method has a wide range of applicability of indoor spaces with complex environments (such as multi-room, room-less, and multi-level building) in a single framework. The proposed method reconstructs the target indoor space as a unique model without segmenting the target indoor space into several sub-spaces. Since both point cloud and trajectory are used, the entire indoor space models, including inter-room and inter-floor connections, can be built in multi-level buildings. However, since reconstruction results rely on the precision of structural points extraction, manual works (e.g., noise removal) can improve the performance of the proposed method.

**Figure 13:**
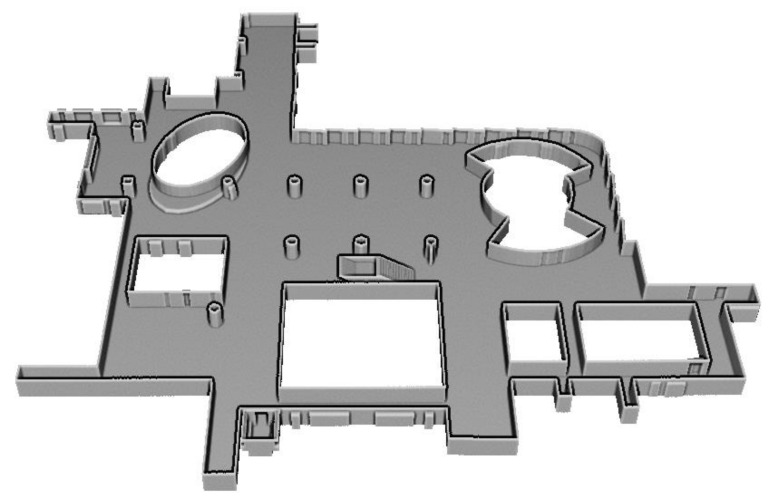
Point cloud of large-scale and room-less indoor space with curved surfaces and cylindrical pillars.

Future work involves the improvement of the proposed method for large-scale and room-less environments as depicted in Figure 13. Additionally, experiments in indoor spaces with curved surfaces and cylindrical pillars will be conducted.

## Figures and Tables

**Table 1:** Parameters for piece-wise planar segments extraction. We extract the planar surfaces iteratively to extract more smaller and detailed planar surfaces. At the first iteration large planar surfaces are extracted, whereas at the second iteration small planar surfaces are extracted.

	First Extraction	Second Extraction
*k*	50	20
$dist_{max}$	2 cm	2 cm
$angle_{max}$	10°	10°
$N_{min}$	200	50

**Table 2:** Environment of indoor spaces used in the experiment.

Dataset	Size W × D × H [m]	#Registered Points	#Trajectory Points	#Rooms	#Floors	#Stairs
A1	18.6 × 8.9 × 3.3	108,508,257	20,972	7	1	0
A2	15.0 × 42.8 × 2.6	32,597,694	89,393	10	1	0
B1	28.1 × 64.5 × 3.5	26,658,402	296	1	1	0
B2	137.2 × 49.1 × 12.8	305,388,028	85,981	1	1	0
C1	13.9 × 10.9 × 10.0	51,600,820	19	11	3	2
C2	13.4 × 12.2 × 7.7	241,610,195	5340	11	3	3
C3	9.0 × 4.8 × 12.2	300,701,197	114,562	11	5	4

**Table 3:** Number of registered point cloud and structural points.

Dataset	#Registered Points	#Structural Points
A1	108,508,257	77,569,453
A2	32,597,694	28,151,560
B1	26,658,402	17,626,644
B2	305,388,028	99,695,180
C1	51,600,820	47,697,095
C2	241,610,195	211,110,747
C3	300,701,197	107,109,986

**Table 4:** Median distance error [cm] and median absolute deviation (MAD) [cm] between structural points and reconstructed mesh.

Dataset	A1	A2	B1	B2	C1	C2	C3
**Median [cm]**	1.53	1.86	1.19	1.41	0.79	1.22	1.21
**MAD [cm]**	1.64	2.92	1.45	3.61	1.76	1.44	1.27
